# Effectiveness of the Adjuvanted Influenza Vaccine in Older Adults at High Risk of Influenza Complications

**DOI:** 10.3390/vaccines9080862

**Published:** 2021-08-05

**Authors:** Constantina Boikos, Mahrukh Imran, Van Hung Nguyen, Thierry Ducruet, Gregg C. Sylvester, James A. Mansi

**Affiliations:** 1 Seqirus Inc., Kirkland, QC H9H 4M7, Canada; Mahrukh.Imran@seqirus.com (M.I.); James.Mansi@modernatx.com (J.A.M.); 2 VHN Consulting, Montreal, QC H2V 3L8, Canada; vhnguyen@vhnconsulting.com (V.H.N.); thierryducruet@vhnconsulting.com (T.D.); 3 Seqirus USA Inc., Summit, NJ 07901, USA; Gregg.Sylvester@Seqirus.com

**Keywords:** MF59-adjuvanted influenza vaccine, relative vaccine effectiveness, high-risk, influenza-related medical encounters, older adults

## Abstract

MF59^®^-adjuvanted trivalent inactivated influenza vaccine (aIIV3) and high-dose trivalent inactivated influenza vaccine (HD-IIV3) elicit an enhanced immune response in older adults compared to standard, quadrivalent inactivated influenza vaccines (IIV4). We sought to determine the relative vaccine effectiveness (rVE) of aIIV3 versus IIV4 and HD-IIV3 in preventing influenza-related medical encounters in this retrospective cohort study involving adults ≥65 years with ≥1 health condition during the 2017–2018 and 2018–2019 influenza seasons. Data were obtained from primary and specialty care electronic medical records linked with pharmacy and medical claims. Adjusted odds ratios (OR) were derived from an inverse probability of treatment-weighted sample adjusted for age, sex, race, ethnicity, geographic region, vaccination week, and health status. rVE was determined using the formula (% rVE = 1 − OR_adjusted_) × 100. Analysis sets included 1,755,420 individuals for the 2017–2018 season and 2,055,012 for the 2018–2019 season. Compared to IIV4, aIIV3 was 7.1% (95% confidence interval 3.3–10.8) and 20.4% (16.2–24.4) more effective at preventing influenza-related medical encounters in the 2017–2018 and 2018–2019 seasons, respectively. Comparable effectiveness was observed with HD-IIV3 across both seasons. Our results support improved effectiveness of aIIV3 vs IIV4 in a vulnerable population of older adults at high risk of influenza and its complications.

## 1. Introduction

Influenza is a major global cause of illness and death, resulting in up to a billion infections, 3–5 million cases of severe disease, and 290,000–650,000 deaths annually [1]. The risk of influenza complications and death may vary depending on factors such as age and the presence of underlying medical conditions [2,3]. The US Advisory Committee on Immunization Practices (ACIP) has identified individuals with chronic pulmonary (including asthma), cardiovascular, renal, hepatic, neurologic, hematologic, or metabolic disorders (including diabetes) as high-risk groups for whom vaccination is “particularly important” and these individuals should be prioritized for immunization when vaccine supply is limited [4]. The risk of serious illness and complications from influenza is even higher in adults 65 years of age and older with underlying medical conditions [5]. Each year in the US, 90% of influenza-related deaths occur in people aged ≥65 years [6,7], primarily as a result of exacerbations of pre-existing cardiovascular and respiratory diseases or due to secondary pneumonia [8,9].

Influenza vaccination is one of the most effective public health measures that has been shown to reduce the burden of influenza disease [10,11]. However, vaccination with standard, nonadjuvanted quadrivalent inactivated influenza vaccines (IIV4) is less effective in adults ≥65 years than in younger adults, largely due to immunosenescence [7,12,13]. This progressive deterioration of the immune system reduces the capacity to respond to novel antigens, such as vaccine antigens, and interferes with long-term immune memory [14,15,16]. Vaccine effectiveness may be further reduced when the circulating virus drifts, leading to changes in the surface proteins of the virus that differ from the vaccine strains, which is most pronounced in the influenza A virus [17]. Two approaches have been developed to provide enhanced protection for older adults: adjuvanted vaccines and high dose vaccines [18,19,20,21]. Vaccines specifically licensed for those ≥65 years of age include the MF59^®^-adjuvanted trivalent inactivated influenza vaccine (aIIV3; Fluad^®^, Seqirus USA Inc., Summit, NJ, USA) and the high-dose nonadjuvanted trivalent inactivated influenza vaccine (HD-IIV3; Fluzone^®^ High-Dose, Sanofi Pasteur Inc., Swiftwater, PA, USA) [18,19]. In clinical studies, aIIV3 also induced cross-reactive antibody production, and as a result, the adjuvanted vaccine may provide heterotypic protection in seasons affected by antigenic drift between circulating virus and vaccine strains [22,23].

Although multiple studies have evaluated the relative vaccine effectiveness (rVE) of aIIV3 in the general population [11,24,25,26,27,28,29,30,31,32,33,34,35], few studies have compared aIIV3 to other influenza vaccines in this particularly vulnerable subsegment of the community, i.e., older adults with underlying medical conditions who are at high risk of influenza and its complications compared with healthy older adults. This analysis was designed to help fill this data gap. The objective of this study was to estimate the rVE of aIIV3 versus unadjuvanted influenza vaccines (IIV4 and HD-IIV3) in preventing influenza-related medical encounters in older adults with medical conditions over two consecutive influenza seasons in the US.

## 2. Materials and Methods

### 2.1. Study Design

This study was part of a larger retrospective cohort study of US adults 65 years of age and older vaccinated with aIIV3, IIV4, or HD-IIV3 that we conducted during the 2017–2018 and the 2018–2019 influenza seasons [34].

### 2.2. Data Sources and Linkage

The analysis was conducted using a dataset integrating patient-level electronic medical records (EMRs) from primary care and specialty clinics (Veradigm Health Insights Ambulatory database) with open and closed claims data (Komodo Healthcare Map), where available. Three national EMR systems form the basis of the integrated dataset, Allscripts Professional, Allscripts Touchworks, and Practice Fusion, and include medical practices of a range of sizes (small practices (1–3 physicians) and medium-sized practices (5–40 physicians)), and integrated delivery networks. The Komodo Healthcare Map consists of anonymized patient-level US pharmacy and medical claims data. The integrated dataset includes data since 2014 for roughly 123 million individuals with representation from all 50 US states and provides comprehensive pharmaceutical, demographic, diagnostic, and healthcare utilization information on patients. The integrated dataset is routinely updated; EMR data are available in almost real-time, while available claims data are available following a lag of several months to allow for adjudication and processing. De-identification and linkage were performed by a third party (Datavant, San Francisco, CA, USA). Two de-identified patient tokens were created from the identifiable information for each patient in both data sources. For patients in both sources with matches on both tokens, a unique patient identifier was created, and the data sources were linked using the common patient identifier. Research staff were not involved in preparation of datasets containing Protected Health Information (PHI) or the actual running of the linkage algorithm. The linked dataset was evaluated and certified for Health Insurance Portability and Accountability Act (HIPAA) compliance.

### 2.3. Exposure Ascertainment

Patient influenza vaccination status (aIIV3, IIV4, HD-IIV3) was ascertained using current procedural terminology (CPT), code for vaccine administered (CVX), and national drug codes (NDCs) in both the EMRs and claims data (Table S1). The main exposure of interest was aIIV3 which was compared separately to both IIV4 and HD-IIV3. The 2 seasonal vaccination intake periods were 1 August 2017 to 28 February 2018, and 1 August 2018 to 28 February 2019. Eligible study participants were classified into 1 of 3 exposure cohorts based on the type of influenza vaccine (aIIV3, IIV4, or HD-IIV3). In addition, a cohort of patients receiving nonadjuvanted, standard dose, trivalent influenza vaccine (IIV3) was also identified. Formulations of IIV4 were first distributed in the US in 2013–2014 and gradually replaced IIV3 in most age groups. Due to limited sample size, IIV3 was not included as a main comparator.

### 2.4. Study Population

The study population included adults ≥65 years of age who had ≥1 medical condition present at the time of recorded immunization with aIIV3, IIV4, or HD-IIV3. Patients were considered fully vaccinated 14 days after recorded receipt of aIIV3, IIV4 or HD-IIV3 to allow for the development of immunity to vaccine-strain influenza viruses. Furthermore, included study subjects must have had at least 1 year of primary care medical history in the EMR platform. Subjects were excluded if they had a record of receiving >1 influenza vaccination during the study season or if they had an influenza-related medical encounter during the study season but prior to the recorded vaccination date. Patients may have been included in the study cohort for one or both influenza seasons under evaluation.

Conditions of interest included chronic pulmonary disease (all conditions), asthma (a subcategory of chronic pulmonary disease that was also evaluated independently), myocardial infarction and/or congestive heart failure, cerebrovascular disease and/or peripheral vascular disease, renal disease, diabetes with chronic complication and/or diabetes without chronic complication, any malignancy and/or metastatic solid tumors, HIV/AIDS, rheumatic disease, mild liver disease and/or moderate or severe liver disease. The ACIP has deemed individuals with “chronic pulmonary (including asthma), cardiovascular (excluding isolated hypertension), renal, hepatic, neurologic, hematologic, or metabolic disorders (including diabetes mellitus)” as well as those immunocompromised due to any cause (such as HIV infection) as high-risk for medical complications attributable to severe influenza [4]. Medical conditions of interest were defined using categories from the Charlson Comorbidity Index (CCI) and coded according to an adaptation of Deyo-Charlson comorbidity score (Table S2) [36]. High-risk categories were not mutually exclusive and individuals could be included in more than 1 category. For instance, if an individual had diabetes and myocardial infarction, they were included separately in each high-risk group.

### 2.5. Outcome Ascertainment

The outcome of interest was a record of an influenza-related medical encounter in both inpatient and outpatient settings. The outcome was ascertained using International Classification of Diseases (ICD)-9-CM and ICD-10-CM codes specific to the diagnosis of influenza disease (Table S3) [37,38]. These codes were identified a priori as the primary outcome of interest [38]. Of note, a broader case definition for “influenza-like illness (ILI)”, corresponding to the Armed Forces Health Surveillance Center’s “Code Set A”, was also evaluated (Table S3).

### 2.6. Covariates

Confounders of the association of interest were identified a priori. Data were ascertained from each subject’s EMR on age (continuous), sex (binary), race (black, white, other), ethnicity (Hispanic and non-Hispanic), US geographic region (South, West, Northeast, Midwest), and health status quantified using individual binary variables for each health condition evaluated in the CCI [36,39]. All covariates were adjusted for in the model, except the binary variable for the specific health condition under evaluation.

### 2.7. Influenza Period

The main observation periods were defined as 1 August 2017 to 19 May 2018 (2017–2018 influenza season) and 1 August 2018 to 18 May 2019 (2018–2019 influenza season). Analyses were conducted separately for each season and the results are reported accordingly.

### 2.8. Statistical Methods

Patient characteristics in the vaccine cohorts during both seasons were evaluated as part of a descriptive analysis. Continuous and categorical variables were reported as mean ± standard deviation and proportional values, respectively. Differences in baseline covariates between the exposure groups were assessed using standardized mean differences (SMD).

Adjusted ORs were calculated from a weighted sample derived using inverse probability of treatment weighting (IPTW) [40]. In the IPTW method, weights are assigned to individuals based on the inverse of their probability of receiving the treatment, as estimated by propensity scores (PS). First, PS were calculated for each cohort defined by a high-risk condition using a multivariable logit model adjusted for age, sex, race, ethnicity, geographic region, week of vaccination, and health status quantified using binary variables that correspond to health conditions identified by the CCI (with the exception of the medical condition under evaluation). PS were then used to create stabilized IPTW. Weights were truncated at the 3rd and 97th percentiles to attenuate any extreme variability from outlier patients. Adjusted ORs were then estimated using a logistic regression model (record of influenza-related medical encounter vs. no influenza-related medical encounter as outcome) in the IPTW-weighted cohort with vaccine type as the predictor for aIIV3 vs. HD-IIV3 and aIIV3 vs. IIV4 comparisons. rVE was calculated as 100 × (1 − OR) and is reported with 95% confidence intervals (CI). Of note, categorical variables with missing or null values in the EMR were classified as ‘not reported/unknown’; missing or out-of-range values were not imputed. Analyses were conducted using SQL and SAS^®^, Version 9.4 (SAS Institute, Cary, NC, USA).

## 3. Results

### 3.1. Study Subjects

**Figure 1:**
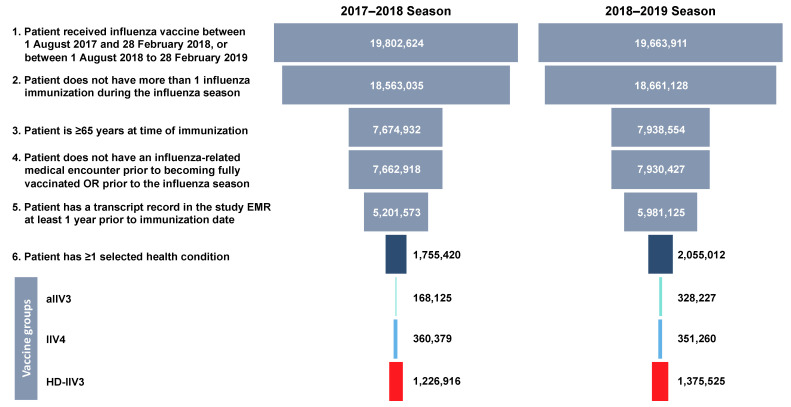
Flow diagram of patient selection process. aIIV3, adjuvanted trivalent inactivated influenza vaccine; EMR, electronic medical record; HD-IIV3, nonadjuvanted high-dose trivalent inactivated influenza vaccine; IIV4, nonadjuvanted quadrivalent inactivated influenza vaccine.

Approximately 20 million individuals were identified from the integrated dataset for each season (Figure 1). The final cohort for the 2017–2018 season included 1,755,420 subjects, of which 168,125 (9.6%) received aIIV3; 360,379 (20.5%) received IIV4; 1,226,916 (69.9%) received HD-IIV3. The 2018–2019 cohort included 2,055,012 patients, divided as follows: aIIV3, 328,227 (16.0%); IIV4, 351,260 (17.1%); HD-IIV3, 1,375,525 (66.9%).

From the 2017–2018 to 2018–2019 season, there was an increase in immunizations with HD-IIV3 and aIIV3 (Figure 1); vaccination with IIV3 decreased over the two seasons (Table S4). Results from the aIIV3 vs IIV3 cohort are reported in the Supplemental Materials.

All vaccine groups were generally comparable in terms of the distributions of age, gender, race, ethnicity, and geographic region (Table 1 and Table S4). During both seasons, most subjects in the vaccine cohorts were female, white, resided in southern US, and had a mean age of ~75 years (Table 1 and Table S4). The most common medical conditions during both seasons across the vaccine cohorts were diabetes, chronic pulmonary disease, peripheral vascular disease, and cancer (Table 1 and Table S4). Moreover, the completeness of covariate information was not observed to differ greatly between the vaccine groups. Standardized mean differences before and after weighting for each of the covariates assessed are shown in Figure S1.

### 3.2. Relative Vaccine Effectiveness

**Figure 2:**
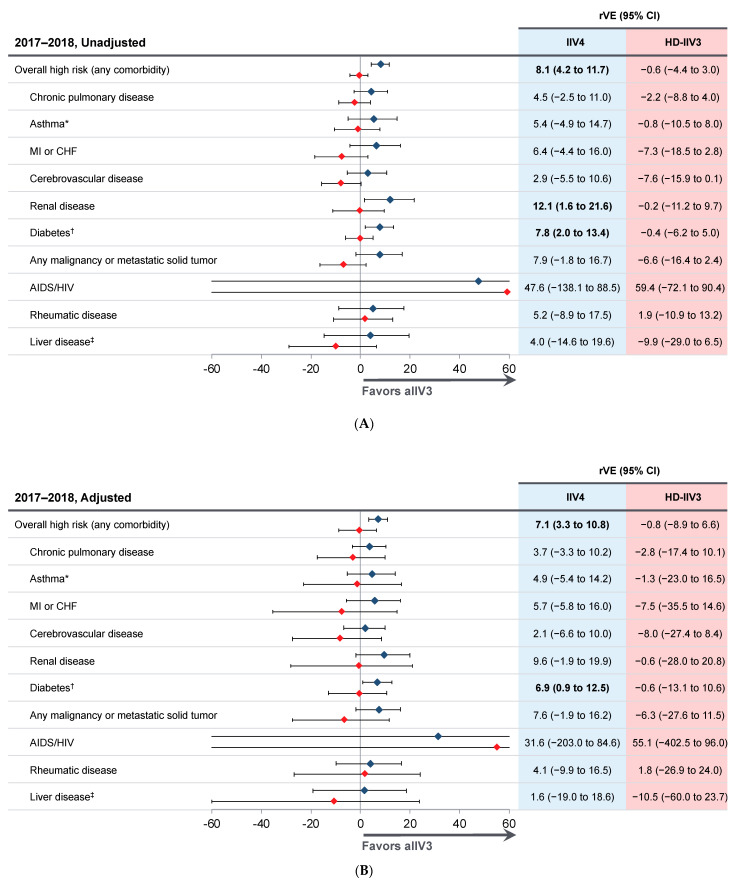

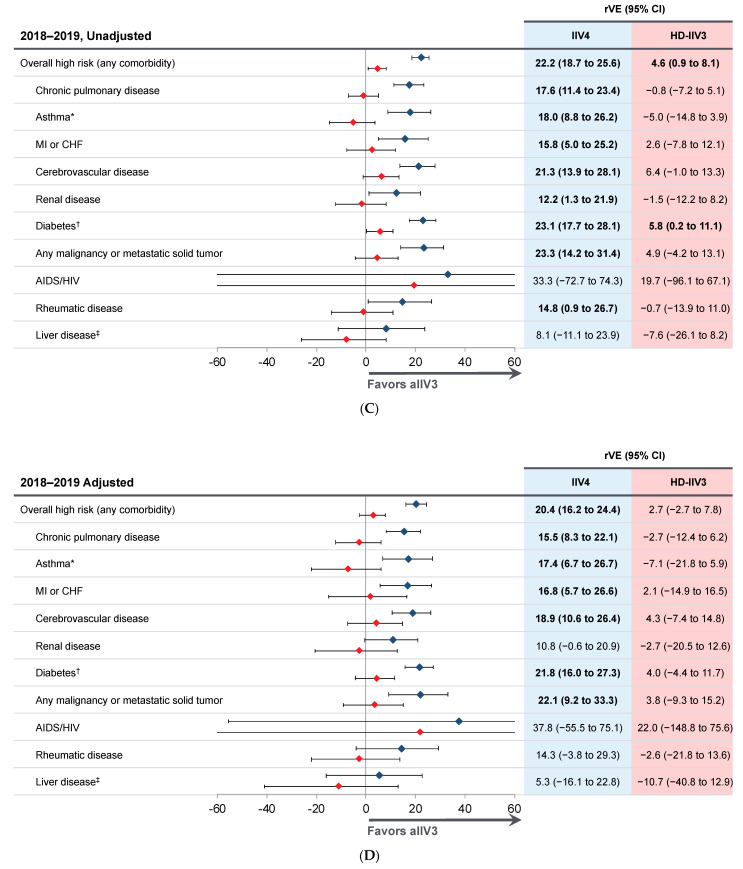
Relative vaccine effectiveness (rVE) of aIIV3 compared with IIV4 (blue) and HD-IIV3 (red) in preventing influenza-related medical encounters (defined by Armed Forces Health Surveillance Center (AFHSC) Code Set B) among adults ≥65 years in the 2017–2018 and 2018–2019 influenza seasons. (**A**) Unadjusted, 2017–2018 season. (**B**) Adjusted, 2017–2018 season. (**C**) Unadjusted, 2018–2019 season. (**D**) Adjusted, 2018–2019 season. Adjusted for age, sex, race, ethnicity, geographic region, week of influenza vaccination, and health status. Boldface indicates statistical significance. aIIV3, adjuvanted trivalent inactivated influenza virus; HD-IIV3, high-dose trivalent inactivated influenza virus; IIV4, quadrivalent inactivated influenza virus. * Subcategory of chronic pulmonary disease. ^†^ With or without chronic complications. ^‡^ Mild, moderate, or severe.

Figure 2 shows the unadjusted and adjusted rVE of aIIV3 vs. IIV4 and aIIV3 vs. HD-IIV3 in both seasons. In 2017–2018, before adjustment, the overall rVE of aIIV3 vs. IIV4 was 8.1% (95% CI 4.2 to 11.7), and of aIIV3 vs. HD-IIV3 was −0.6% (95% CI −4.4 to 3.0). In 2018-2019, the unadjusted rVEs were 22.2% (95% CI 18.7 to 25.6) and 4.6% (95% CI 0.9 to 8.1) vs. IIV4 and HD-IIV3, respectively. After adjustment, the overall rVE of aIIV3 vs. was 7.1% (95% CI 3.3 to 10.8) vs. IIV4 and −0.8% (95% CI −8.9 to 6.6) vs. HD-IIV3 in 2017-2018, and in 2018-2019 the rVE was 20.4% (95% CI 16.2 to 24.4) and 2.7% (95% CI −2.7 to 7.8) vs. IIV4 and HD-IIV3, respectively. In 2017-2018, adjusted rVE values for individual comorbidities were not statistically significant except for the aIIV3 vs. IIV4 comparison for diabetes (Figure 2B). In 2018−2019, rVEs were statistically significant in comparisons of aIIV3 vs. IIV4 for all high-risk conditions except renal disease, HIV/AIDS, rheumatic disease, and liver disease (Figure 2D). Comparisons between aIIV3 and HD-IIV3 were not statistically significant in either season (Figure 2). Adjusted rVEs for comparisons between aIIV3 and IIV3 appear in Table S5, and Table S6 displays the rVE of aIIV3 against IIV4, HD-IIV3, and IIV3 using the broader case definition (AFHSC Code Set A).

## 4. Discussion

Medical conditions including chronic cardiopulmonary and respiratory diseases have been established as risk factors for influenza and influenza-associated complications [41]. The risk of serious illness and complications from influenza is even more pronounced in older adults with underlying medical conditions due to immunosenescence [2,3]. A previous study has shown that the MF59^®^ adjuvant enhances protection against influenza by increasing both the magnitude and breadth of the immune response [35].

Over both the 2017–2018 and 2018–2019 seasons, aIIV3 demonstrated significantly improved clinical benefit compared with IIV4 and HD-IIV3 in our study of the general population of older adults ≥65 years of age [34]. However, the relative effectiveness of influenza vaccines among individuals with high-risk medical conditions had not been extensively evaluated, leaving potential uncertainty around the relative benefits of specific influenza vaccines in these important high-risk population subgroups that are often excluded from randomized controlled trials [41]. The presence of underlying medical conditions may affect immunogenicity of the influenza vaccine which may in turn impact VE estimates, particularly in an age group impacted by immunosenescence [42]. This is one of the first studies evaluating the rVE of aIIV3 vs IIV4 and HD-IIV3 specifically in older adults with underlying medical conditions, who are at high risk of influenza and its complications.

Adjusted analyses from this study showed that subjects who received aIIV3 had significantly fewer influenza-related medical encounters compared with subjects vaccinated with IIV4. In the 2017–2018 season, a statistically significant benefit of aIIV3 compared to IIV4 was observed among the overall high-risk study population as well as in patients with diabetes. Similarly, in the 2018–2019 season, a statistically significant benefit of aIIV3 compared to IIV4 was observed in the overall high-risk study population, as well as in subgroups of individuals with chronic pulmonary disease (including asthma), myocardial infarction and/or congestive heart failure, cerebrovascular disease and/or peripheral vascular disease, diabetes, any malignancy and/or metastatic solid tumors, or rheumatic disease. These results are consistent with our larger retrospective cohort study evaluating over 11 million vaccinated individuals ≥65 years, in which aIIV3 was more effective than IIV4 in reducing influenza-related medical encounters [34]. In the comparison with HD-IIV3, the point estimates hovered around the null and were not statistically significant, precluding definitive conclusions and suggesting comparable effectiveness between both enhanced vaccines in these high-risk population groups.

The 2017–2018 season was a “high severity” season dominated by circulating A(H3N2) influenza viruses with some B/Yamagata circulation, and overall vaccine effectiveness was estimated to be 17% (95% CI −14 to 39) in subjects aged ≥65 years [43,44]. The 2018–2019 season, which was considered “moderate severity”, was dominated by 1 wave of influenza A(H1N1) from October 2018 to mid-February 2019 and a second wave of influenza A(H3N2) from February through May 2019. During this season, the overall vaccine effectiveness in subjects ≥65 years was 12% (95% CI −31 to 40) [45]. Although aIIV3 demonstrated consistently higher relative vaccine effectiveness compared to IIV4 over both seasons in the overall high-risk study population, it should also be noted that the relative effectiveness of aIIV3 vs. IIV4 was higher in the 2018–2019 season than in the 2017–2018 season. The inclusion of the additional B strain (B-Yamagata) in IIV4 compared to aIIV3 may have attenuated the relative benefit of the adjuvant in the 2017–2018 season, since more than 20% of circulating viruses were B-Yamagata [43]. Differences in the impact of drift in these two seasons may have also contributed to the adjuvant providing a stronger benefit relative to an unadjuvanted vaccine in one vs. the other season [22,43,45]. The 2017–2018 A(H3N2) vaccine virus was a 3C.2a clade virus, as were the majority of circulating viruses [43]. The 2018–2019 A(H3N2) vaccine virus was a 3C.2a1 clade virus, whereas the majority of circulating A(H3N2) viruses were 3C.3a (73.9%). Antigenic testing showed that 99.4% of 3C.3a viruses were not well inhibited by the 2018–2019 A(H3N2) vaccine virus [45]. In the 2018–2019 season, the majority of circulating A(H1N1) viruses were clade 6B.1 viruses with a S183P substitution, which showed reduced titers in post-vaccination human antisera testing compared to the vaccine virus (clade 6B.1 with no S183P substitution) [46]. Viral characterization data suggest that there was not any substantial drift during the 2017–2018 season, whereas the majority of circulating A(H3N2) and A(H1N1) viruses may have been antigenically drifted during the 2018–2019 season. While a clinical benefit was seen in both seasons, the increased magnitude and breadth of immune response offered by MF59 may explain the greater clinical benefit of aIIV3 compared with IIV4 in the 2018–2019 season compared to 2017–2018 [35].

Key strengths of this study included the use of an integrated database linking both EMR and claims data, which permitted evaluation of a large cohort of older adults with underlying medical conditions, a population that is often not included in randomized controlled trials (RCTs). The comprehensive nature of the data also permitted adjustment of well-established confounders using IPTW, a robust confounder adjustment methodology. Exposure, outcome, and covariate information were determined retrospectively from patient records in exactly the same manner for all exposure cohorts, limiting the possibility of differential misclassification of these elements. The database allowed individuals with underlying medical conditions to be identified using validated ICD-9/10 algorithms for CCI categories [36].

In addition to these strengths, several limitations should be noted. First, influenza infection was not laboratory confirmed. However, a descriptive evaluation of the overlap between the incidence of CDC-reported, laboratory-confirmed influenza and the incidence of influenza-related medical encounters (AFHSC Code Set B) in the integrated dataset was conducted in our larger retrospective cohort study [34]. An observed concordance between trends supports the use of the diagnostic AFHSC Code Set B in evaluations of influenza. Next, although the overall study population was large, stratification by specific medical conditions resulted in small sample sizes for subgroups—such as those with HIV/AIDS—which limited statistical power. Furthermore, the use of diagnostic codes to identify high-risk health conditions does not necessarily permit differentiation of the level of severity or immunosuppression within each specific condition, and the influence of these factors on vaccine effectiveness was not assessed. Moreover, the analysis did not specifically adjust for frailty, which is associated with uptake of enhanced vaccines and increased risk of influenza complications, which may confound rVE estimates. Additionally, the study cohort, which included subjects for whom at least some pharmacy and medical claims data were available, was thus limited to insured individuals but did not require healthcare resource utilization beyond the index vaccination. Lastly, this study has limitations inherent to observational studies: These studies cannot demonstrate causality, but can rather provide evidence for, and show the strength of, an association. As vaccination was not randomly assigned, despite robust IPTW adjustment, residual confounding may still have an impact on estimates of rVE in this analysis.

## 5. Conclusions

Vaccination represents the most effective public health intervention for the prevention of seasonal influenza infection, hospitalization, and mortality. International guidelines and preventive policies regarding influenza vaccination are primarily focused on protecting high-risk individuals by vaccinating them or those who could infect them [4,47]. This study demonstrates that, in a cohort of high-risk adults ≥65 years of age, patients with a record of aIIV3 had statistically significantly fewer influenza-related medical encounters compared to individuals with a record of IIV4 in the 2017–2018 and 2018–2019 influenza seasons in the US. Comparable effectiveness with HD-IIV3 was observed. By utilizing EMRs linked to claims data, we were able to evaluate a large study population and healthcare settings that reflect real-world conditions. Our findings are consistent with previously published studies evaluating the relative efficacy of aIIV3 compared to standard vaccines [11,24,25,26,27,28,29,30,31,32,33,34,35].

## Figures and Tables

**Table 1:** Subject demographics at baseline.

	2017–2018 Season			2018–2019 Season		
Characteristic	aIIV3 (*n* = 168,125)	IIV4 (*n* = 360,379)	HD-IIV3 (*n* = 1,226,916)	aIIV3 (*n* = 328,227)	IIV4 (*n* = 351,260)	HD-IIV3 (*n* = 1,375,525)
Mean age, years ± SD	75.6 ± 6.7	74.9 ± 7.1	75.8 ± 6.8	75.7 ± 6.8	74.9 ± 7.2	75.8 ± 6.9
Female, *n* (%)	93,970 (56)	202,670 (56)	681,260 (56)	182,214 (56)	198,131 (56)	767,661 (56)
Race, *n* (%)						
White	112,077 (67)	230,571 (64)	830,987 (68)	215,363 (66)	207,481 (59)	896,117 (65)
Black	9050 (5)	24,707 (7)	71,805 (6)	16,043 (5)	26,427 (8)	81,066 (6)
Other	11,669 (7)	38,438 (11)	90,175 (7)	25,660 (8)	37,301 (11)	109,393 (8)
Not reported	35,329 (21)	66,663 (18)	233,949 (19)	71,161 (22)	80,051 (23)	288,949 (21)
Ethnicity, *n* (%)						
Hispanic	7982 (5)	24,334 (7)	45,310 (4)	14,059 (4)	27,732 (8)	52,392 (4)
Non-Hispanic	136,560 (81)	289,830 (80)	1,009,570 (82)	267,577 (82)	280,017 (80)	1,123,324 (82)
Not reported	23,583 (14)	46,215 (13)	172,036 (14)	46,591 (14)	43,511 (12)	199,809 (15)
Geographic region, *n* (%)						
Northeast	22,459 (13)	61,643 (17)	251,559 (21)	48,881 (15)	63,524 (18)	254,344 (18)
Midwest	17,365 (10)	69,256 (19)	276,944 (23)	45,554 (14)	65,034 (19)	306,667 (22)
South	106,500 (63)	138,853 (39)	479,837 (39)	192,007 (58)	138,857 (40)	541,477 (39)
West	18,681 (11)	85,298 (24)	202,316 (16)	35,671 (11)	76,013 (22)	254,799 (19)
Not reported/other	3120 (2)	5329 (1)	16,260 (1)	6114 (2)	7832 (2)	18,238 (1)
High-risk health condition						
Chronic pulmonary disease	46,020 (27)	101,502 (28)	341,912 (28)	90,221 (27)	102,422 (29)	394,723 (29)
Myocardial infarction	8101 (5)	18,783 (5)	62,436 (5)	15,953 (5)	17,182 (5)	68,833 (5)
Congestive heart failure	12,343 (7)	34,350 (10)	111,431 (9)	24,036 (7)	33,294 (9)	122,219 (9)
Cerebrovascular disease	19,562 (12)	44,664 (12)	157,325 (13)	40,988 (12)	46,549 (13)	185,964 (14)
Peripheral vascular disease	23,331 (14)	59,620 (17)	186,771 (15)	45,314 (14)	55,339 (16)	206,224 (15)
Renal disease	19,327 (11)	50,437 (14)	161,107 (13)	39,964 (12)	52,760 (15)	197,466 (14)
Diabetes not chronic	40,823 (24)	107,093 (30)	329,154 (27)	78,948 (24)	102,092 (29)	362,658 (26)
Diabetes chronic	62,692 (37)	134,253 (37)	422,923 (34)	124,193 (38)	143,480 (41)	494,782 (36)
Any malignancy	26,010 (15)	50,567 (14)	198,815 (16)	55,147 (17)	50,749 (14)	230,268 (17)
Metastatic tumor	8493 (5)	13,198 (4)	48,364 (4)	14,583 (4)	12,154 (3)	52,711 (4)
AIDS/HIV	266 (0)	772 (0)	1420 (0)	577 (0)	967 (0)	2012 (0)
Rheumatic disease	12,463 (7)	25,370 (7)	89,461 (7)	23,753 (7)	23,121 (7)	96,184 (7)
Mild liver disease	7875 (5)	21,202 (6)	65,472 (5)	15,992 (5)	19,440 (6)	73,602 (5)
Liver disease	403 (0)	1233 (0)	3645 (0)	752 (0)	1056 (0)	3804 (0)
Hemiplegia or paraplegia	884 (1)	3146 (1)	8163 (1)	1836 (1)	3448 (1)	10,088 (1)
Dementia	5474 (3)	15,486 (4)	45,716 (4)	9766 (3)	16,704 (5)	53,106 (4)
Peptic ulcer disease	4548 (3)	9666 (3)	35,361 (3)	9220 (3)	9080 (3)	38,681 (3)
Charlson comorbidity index, mean ± SD	2.2 ± 1.5	2.2 ± 1.4	2.1 ± 1.5	2.2 ± 1.5	2.2 ± 1.4	2.2 ± 1.4

## Data Availability

The datasets used in this study are privately licensed and are not available in order to maintain patient privacy.

## Supplementary Materials

The following are available online at https://www.mdpi.com/article/10.3390/vaccines9080862/s1, Table S1: List of CPT, CVX, and NDC codes used to identify influenza vaccines from the Veradigm EMR dataset, Table S2: ICD-9-CM and ICD-10 Coding Algorithms for Charlson Comorbidities, Table S3: Influenza and influenza-like illness code set definitions, Table S4: Subject demographics at baseline in the IIV3 cohort, Table S5: Adjusted rVE of aIIV3 versus comparators using AFHSC Code Set B to define influenza-related medical encounters in the 2017–2018 and 2018–2019 influenza seasons by age group, Table S6: Adjusted rVE of aIIV3 versus comparators using AFHSC Code Set A to define influenza in the 2017–2018 and 2018–2019 influenza seasons by age group, Figure S1: Standardized mean differences between aIIV3 and comparators.
