# Supplementary material for: Effectiveness of the Adjuvanted Influenza Vaccine in Older Adults at High Risk of Influenza Complications
Source: Vaccines (Basel). 2021 Aug 5;9(8):862. doi: 10.3390/vaccines9080862 (PMC8402367; doi:10.3390/vaccines9080862)
Supplement: Supplementary file 1 [file vaccines-09-00862-s001.zip › vaccines-1281392-supplementary.pdf]

**SUPPLEMENTAL MATERIAL**

# Effectiveness of Adjuvanted Influenza Vaccine in Older Adults at High Risk of Influenza Complications

Constantina Boikos, Mahrukh Imran, Van Hung Nguyen, Thierry Ducruet, Gregg C. Sylvester, James A. Mansi

**Table S1.** List of CPT, CVX, and NDC codes used to identify influenza vaccines from the Veradigm EMR dataset.

| Influenza vaccine type | CPT | CVX | NDC |
|---|---|---|---|
| aIIV3 | 90653 | 168 | 66521-0000-01, 70461-0001-01, 70461-0002-01, 70461-0018-03, 70461-0019-03, 66521-0000-11, 70461-0001-11, 70461-0002-11, 70461-0018-04, 70461-0019-04 |
| HD-IIV3 | 90662 | 135 | 49281-0389-65, 49281-0391-65, 49281-0393-65, 49281-0395-65, 49281-0397-65, 49281-0399-65, 49281-0401-65, 49281-0403-65, 49281-0405-65, 49281-0393-88, 49281-0395-88, 49281-0397-88, 49281-0399-88, 49281-0401-88, 49281-0403-88, 49281-0405-88 |
| IIV4 | 90685, 90686, 90687, 90688, 90630 | 158, 150, 161 | 33332-0219-20, 33332-0316-01, 33332-0317-01, 33332-0318-01, 33332-0319-01, 33332-0416-10, 33332-0417-10, 33332-0418-10, 33332-0419-10, 33332-0219-21, 33332-0316-02, 33332-0317-02, 33332-0318-02, 33332-0319-02, 33332-0416-11, 33332-0417-11, 33332-0418-11, 33332-0419-11, 58160-0896-52, 58160-0900-52, 58160-0903-52, 58160-0905-52, 58160-0907-52, 58160-0898-52, 58160-0901-52, 58160-0896-41, 58160-0900-41, 58160-0903-41, 58160-0905-41, 58160-0907-41, 58160-0898-41, 58160-0901-41, 19515-0891-11, 19515-0894-52, 19515-0895-11, 19515-0897-11, 19515-0898-11, 19515-0901-52, 19515-0903-11, 19515-0906-52, 19515-0908-52, 19515-0896-11, 19515-0900-11, 19515-0909-52, 19515-0912-52, 19515-0891-01, 19515-0894-41, 19515-0895-01, 19515-0897-01, 19515-0898-01, 19515-0901-41, 19515-0903-01, 19515-0906-41, 19515-0908-41, 19515-0896-01, 19515-0900-01, 19515-0909-41, 19515-0912-41, 49281-0413-10, 49281-0413-50, 49281-0414-10, 49281-0414-50, 49281-0415-10, 49281-0416-10, 49281-0416-50, 49281-0417-10, 49281-0417-50, 49281-0418-10, 49281-0418-50, 49281-0419-10, 49281-0419-50, 49281-0513-25, 49281-0514-25, 49281-0516-25, 49281-0517-25, 49281-0518-25, 49281-0519-25, 49281-0621-15, 49281-0625-15, 49281-0627-15, 49281-0629-15, 49281-0631-15, 49281-0413-58, 49281-0413-88, 49281-0414-58, 49281-0414-88, 49281-0415-58, 49281-0416-58, 49281-0416-88, 49281-0417-58, 49281-0417-88, 49281-0418-58, 49281-0418-88, 49281-0419-58, 49281-0419-88, 49281-0513-00, 49281-0514-00, 49281-0516-00, 49281-0517-00, 49281-0518-00, 49281-0519-00, 49281-0621-78, 49281-0625-78, 49281-0627-78, 49281-0629-78, 49281-0631-78 |
| IIV3 | 90656, 90658 | 141, 140 | 33332-0010-01, 33332-0013-01, 33332-0014-01, 33332-0015-01, 33332-0016-01, 33332-0017-01, 33332-0018-01, 33332-0110-10, 33332-0113-10, 33332-0114-10, 33332-0115-10, 33332-0116-10, 33332-0117-10, 33332-0118-10, 33332-0013-02, 33332-0014-02, 33332-0015-02, 33332-0016-02, 33332-0017-02, 33332-0018-02, 33332-0113-11, 33332-0114-11, |

| Influenza vaccine type | CPT | CVX | NDC |
|---|---|---|---|
| | | | 33332-0115-11, 33332-0116-11, 33332-0117-11, 33332-0118-11 |

aIIV3, adjuvanted trivalent inactivated influenza vaccine; CPT, current procedural terminology; CVX, code for vaccine administered; HD-IIV3, high-dose nonadjuvanted trivalent inactivated influenza vaccine; IIV3, nonadjuvanted egg-derived trivalent inactivated influenza vaccine; IIV4, nonadjuvanted egg-derived quadrivalent inactivated influenza vaccine; NDC, national drug code.

**Table S2. ICD-9-CM and ICD-10 Coding Algorithms for Charlson Comorbidities**

| Charlson comorbidity category | Deyo's ICD-9-CM + | ICD-10 * | Enhanced ICD-9-CM * |
|---|---|---|---|
| Myocardial infarction | 410.x, 412.x | I21.x, I22.x, I25.2 | 410.x, 412.x |
| Congestive heart failure | 428.x | I09.9,I11.0, I13.0, I13.2, I25.5, I42.0, I42.5-I42.9, I43.x, I50.x, P29.0 | 398.91, 402.01, 402.11, 402.91, 404.01, 404.03, 404.11, 404.13, 404.91, 404.93, 425.4-425.9, 428.x |
| Peripheral vascular disease | 443.9, 441.x, 785.4, V43.4 Procedure 38.48 | I70.x, I71.x, I73.1, I73.8, I73.9, I77.1, I79.0, I79.2, K55.1, K55.8, K55.9, Z95.8, Z95.9 | 093.0, 437.3, 440.x, 441.x, 443.1-443.9, 447.1, 557.1, 557.9, V43.4 |
| Cerebrovascular disease | 430.x-438.x | G45.x, G46.x, H34.0, I60.x-I69.x | 362.34, 430.x-438.x |
| Dementia | 290.x | F00.x-F03.x, F05.1, G30.x, G31.1 | 290.x, 294.1, 331.2 |
| Chronic pulmonary Disease | 490.x-505.x, 506.4 | I27.8, I27.9, J40.x-J47.x, J60.x-J67.x, J68.4, J70.1, J70.3 | 416.8, 416.9, 490.x-505.x, 506.4, 508.1, 508.8 |
| Rheumatic disease | 710.0, 710.1, 710.4, 714.0-714.2, 714.81, 725.x | M05.x, M06.x, M31.5, M32.x-M34.x, M35.1, M35.3, M36.0 | 446.5, 710.0-710.4, 714.0-714.2, 714.8, 725.x |
| Peptic ulcer disease | 531.x-534.x | K25.x-K28.x | 531.x-534.x |
| Mild liver disease | 571.2, 571.4-571.6 | B18.x, K70.0-K70.3, K70.9, K71.3-K71.5, K71.7, K73.x, K74.x, K76.0, K76.2-K76.4, K76.8, K76.9, Z94.4 | 070.22, 070.23, 070.32, 070.33, 070.44, 070.54, 070.6, 070.9, 570.x, 571.x, 573.3, 573.4, 573.8, 573.9, V42.7 |
| Diabetes without chronic complication | 250.0-250.3, 250.7 | E10.0, E10.l, E10.6, E10.8, E10.9, E11.0, E11.1, E11.6, E11.8, E11.9, E12.0, E12.1, E12.6, E12.8, E12.9, E13.0, E13.1, E13.6, E13.8, E13.9, E14.0, E14.1, E14.6, E14.8, E14.9 | 250.0-250.3, 250.8, 250.9 |
| Diabetes with chronic | 250.4-250.6 | E10.2-E10.5, E10.7, E11.2-E11.5, | 250.4-250.7 |

| Charlson comorbidity category | Deyo's ICD-9-CM + | ICD-10 * | Enhanced ICD-9-CM * |
|---|---|---|---|
| complication | | E11.7, E12.2-E12.5, E12.7, E13.2-E13.5, E13.7, E14.2-E14.5, E14.7 | |
| Hemiplegia or paraplegia | 344.1, 342.x | G04.1, G11.4, G80.1, G80.2, G81.x, G82.x, G83.0-G83.4, G83.9 | 334.1, 342.x, 343.x, 344.0-344.6, 344.9 |
| Renal disease | 582.x, 583-583.7, 585.x, 586.x, 588.x | I12.0, I13.1, N03.2-N03.7, N05.2-N05.7, N18.x, N19.x, N25.0, Z49.0-Z49.2, Z94.0, Z99.2 | 403.01, 403.11, 403.91, 404.02, 404.03, 404.12, 404.13, 404.92, 404.93, 582.x, 583.0-583.7, 585.x, 586.x, 588.0, V42.0, V45.1, V56.x |
| Any malignancy, including lymphoma and leukemia, except malignant neoplasm of skin | 140.x-172.x, 174.x-195.8, 200.x-208.x | C00.x-C26.x, C30.x-C34.x, C37.x-C41.x, C43.x, C45.x-C58.x, C60.x-C76.x, C81.x-C85.x, C88.x, C90.x-C97.x | 140.x-172.x, 174.x-195.8, 200.x-208.x, 238.6 |
| Moderate or severe liver disease | 456.0-456.21, 572.2-572.8 | I85.0, I85.9, I86.4, I98.2, K70.4, K71.1, K72.1, K72.9, K76.5, K76.6, K76.7 | 456.0-456.2, 572.2-572.8 |
| Metastatic solid tumor | 196.x-199.1 | C77.x-C80.x | 196.x-199.x |
| AIDS/HIV | 042.x-044.x | B20.x-B22.x, B24.x | 042.x-044.x |

**Table S3. Influenza and Influenza-Like Illness Code Set Definitions**

Code Set A was intended for general surveillance efforts and includes the following International Classification of Disease (ICD) codes[1]:

| Condition: Influenza-like Illness | |
|---|---|
| **ICD-10-CM Codes** | **ICD-9-CM Codes** |
| B97.89 (other viral agents as the cause of diseases classified elsewhere) | 079.99 (unspecified viral infection) |
| H66.9 (otitis media, unspecified) | 382.9 (unspecified otitis media) |
| H66.90 (otitis media, unspecified, unspecified ear) | |
| H66.91 (otitis media, unspecified, right ear) | |
| H66.92 (otitis media, unspecified, left ear) | |
| H66.93 (otitis media, unspecified, bilateral ear) | |
| J00 (acute nasopharyngitis; common cold) | 460 (acute nasopharyngitis) |
| J01.9 (acute sinusitis, unspecified) | 461.9 (acute sinusitis, unspecified) |
| J01.90 (acute sinusitis, unspecified) | |
| J06.9 (acute upper respiratory infection, unspecified) | 465.8 (acute upper respiratory infections, other multiple sites) |
| | 465.9 (acute upper respiratory infections, unspecified sites) |
| J09 (influenza due to certain identified influenza viruses) | — |
| J09.X (influenza due to identified novel influenza A viruses) | — |
| J09.X1 (influenza due to identified novel influenza A virus with pneumonia) | 488.0 (influenza due to identified avian influenza virus) |
| | 488.01 (influenza due to identified avian influenza virus with pneumonia) |
| | 488.8 (influenza due to novel influenza A) |
| | 488.81 (influenza due novel influenza A with pneumonia) |
| J09.X2 (influenza due to identified novel influenza A virus with other respiratory manifestations) | 488.02 (influenza due to identified avian influenza virus with other respiratory manifestations) |
| | 488.82 (influenza due to novel influenza A with other respiratory manifestations) |
| J09.X3 (influenza due to identified novel influenza A virus with gastrointestinal manifestations) | 488.09 (influenza due to identified avian influenza virus with other manifestations) |
| | 488.1 (influenza due to 2009 H1N1 influenza virus) |
| | 488.19 (influenza due to identified 2009 H1N1 influenza virus with other manifestations) |

| Condition: Influenza-like Illness | |
|---|---|
| **ICD-10-CM Codes** | **ICD-9-CM Codes** |
| J09.X9 (influenza due to identified novel influenza A virus with other manifestations) | 488.89 (influenza due to novel influenza A with other manifestations) |
| J10 (influenza due to other identified influenza viruses) | — |
| J10.0 (influenza due to identified novel influenza A viruses) | — |
| J10.00 (influenza due to other identified influenza virus with unspecified type of pneumonia) | 487.0 (influenza with pneumonia) |
| J10.01 (influenza due to other identified influenza virus with the same other identified influenza virus pneumonia) | 487.1 (influenza with other respiratory manifestations) |
| J10.08 (influenza due to other identified influenza virus with other specified pneumonia) | 487.0 (above) |
| | 488.11 (influenza due to identified 2009 H1N1 influenza virus with pneumonia) |
| J10.1 (influenza due to other identified influenza virus with other respiratory manifestations) | 487.1 (above) |
| | 488.12 (influenza due to identified 2009 H1N1 influenza virus with other respiratory manifestations) |
| J10.2 (influenza due to other identified influenza virus with gastrointestinal manifestations) | 487.8 (influenza with other manifestations) |
| J10.8 (influenza due to other identified influenza virus with other manifestations) | |
| J10.81 (influenza due to other identified influenza virus with other manifestations with encephalopathy) | |
| J10.82 (influenza due to other identified influenza virus with other manifestations with myocarditis) | |
| J10.83 (influenza due to other identified influenza virus with other manifestations with otitis media) | |
| J10.89 (influenza due to other identified influenza virus with other manifestations) | |
| J11 (influenza due to unidentified influenza virus) | — |
| J11.0 (influenza due to unidentified influenza virus with pneumonia) | — |
| J11.00 (influenza due to unidentified influenza virus with unspecified type of pneumonia) | 487.0 (above) |

| Condition: Influenza-like Illness | |
|---|---|
| **ICD-10-CM Codes** | **ICD-9-CM Codes** |
| J11.08 (influenza due to unidentified influenza virus with specified pneumonia) | |
| J11.1 (influenza due to unidentified influenza virus with other respiratory manifestations) | 487.1 (above) |
| J11.2 (influenza due to unidentified influenza virus with gastrointestinal manifestations) | 487.8 (above) |
| J11.8 (influenza due to unidentified influenza virus with other manifestations) | |
| J11.81 (influenza due to unidentified influenza virus with encephalopathy) | |
| J11.82 (influenza due to unidentified influenza virus with myocarditis) | |
| J11.83 (influenza due to unidentified influenza virus with otitis media)) | |
| J11.89 (influenza due to unidentified influenza virus with other manifestations) | |
| J12.89 (other viral pneumonia) | 487.0 (above) |
| J12.9 (viral pneumonia, unspecified) | |
| J18 (pneumonia, unspecified organism) | 486 (pneumonia, organism unspecified) |
| J18.1 (lobar pneumonia, unspecified organism) | |
| J18.8 (other pneumonia, unspecified organism) | |
| J18.9 (pneumonia, unspecified organism) | |
| J20.9 (acute bronchitis, unspecified) | 466.0 (acute bronchitis) |
| R05 (cough) | 786.2 (cough) |
| | 780.6 (fever and other physiologic disturbances of temperature regulation) |
| J40 (bronchitis, not specified as acute or chronic) | 490 (bronchitis, not specified as acute or chronic) |
| R50.9 (fever, unspecified) | 780.60 (fever, unspecified) |

Code Set B was intended for specific epidemiologic investigations and includes the following ICD codes[1]:

| Condition: Influenza | |
|---|---|
| **ICD-10-CM Codes** | **ICD-9-CM Codes** |
| J09 (influenza due to certain identified influenza viruses) | — |
| J09.X1 (influenza due to identified novel influenza A virus with pneumonia) | 488.0 (influenza due to identified avian influenza virus) |
| | 488.01 (influenza due to identified avian influenza virus with pneumonia) |
| | 488.8 (influenza due to novel influenza A) |

| Condition: Influenza | |
|---|---|
| **ICD-10-CM Codes** | **ICD-9-CM Codes** |
| | 488.81 (influenza due novel influenza A with pneumonia) |
| J09.X2 (influenza due to identified novel influenza A virus with other respiratory manifestations) | 488.02 (influenza due to identified avian influenza virus with other respiratory manifestations) |
| | 488.82 (influenza due to novel influenza A with other respiratory manifestations) |
| J09.X3 (influenza due to identified novel influenza A virus with gastrointestinal manifestations) | 488.09 (influenza due to identified avian influenza virus with other manifestations) |
| | 488.1 (influenza due to 2009 H1N1 influenza virus) |
| | 488.19 (influenza due to identified 2009 H1N1 influenza virus with other manifestations) |
| J09.X9 (influenza due to identified novel influenza A virus with other manifestations) | 488.89 (influenza due to novel influenza A with other manifestations) |
| J10 (influenza due to other identified influenza viruses) | — |
| J10.0 (influenza due to identified novel influenza A viruses) | — |
| J10.00 (influenza due to other identified influenza virus with unspecified type of pneumonia) | 487.0 (influenza with pneumonia) |
| J10.01 (influenza due to other identified influenza virus with the same other identified influenza virus pneumonia) | 487.1 (influenza with other respiratory manifestations) |
| J10.08 (influenza due to other identified influenza virus with other specified pneumonia) | 487.0 (above) |
| | 488.11 (influenza due to identified 2009 |
| | H1N1 influenza virus with pneumonia) |
| J10.1 (influenza due to other identified influenza virus with other respiratory manifestations) | 487.1 (above) |
| | 488.12 (influenza due to identified 2009 |
| | H1N1 influenza virus with other respiratory manifestations) |
| J10.2 (influenza due to other identified influenza virus with gastrointestinal manifestations) | 487.8 (influenza with other manifestations) |
| J10.8 (influenza due to other identified influenza virus with other manifestations) | |

| Condition: Influenza | |
|---|---|
| **ICD-10-CM Codes** | **ICD-9-CM Codes** |
| J10.81 (influenza due to other identified influenza virus with other manifestations with encephalopathy) | |
| J10.82 (influenza due to other identified influenza virus with other manifestations with myocarditis) | |
| J10.83 (influenza due to other identified influenza virus with other manifestations with otitis media) | |
| J10.89 (influenza due to other identified influenza virus with other manifestations) | |
| J11 (influenza due to unidentified influenza virus) | — |
| J11.0 (influenza due to unidentified influenza virus with pneumonia) | — |
| J11.00 (influenza due to unidentified influenza virus with unspecified type of pneumonia) | 487.0 (above) |
| J11.08 (influenza due to unidentified influenza virus with specified pneumonia) | |
| J11.1 (influenza due to unidentified influenza virus with other respiratory manifestations) | 487.1 (above) |
| J11.2 (influenza due to unidentified influenza virus with gastrointestinal manifestations) | 487.8 (above) |
| J11.8 (influenza due to unidentified influenza virus with other manifestations) | |
| J11.81 (influenza due to unidentified influenza virus with encephalopathy) | |
| J11.82 (influenza due to unidentified influenza virus with myocarditis) | |
| J11.83 (influenza due to unidentified influenza virus with otitis media)) | |
| J11.89 (influenza due to unidentified influenza virus with other manifestations) | |

Code Set B includes only those codes with >75% influenza positivity for the matched laboratory test, which limits the number of false positive results.[2,3]

**References**


1. Armed Forces Health Surveillance Center (AFHSC). AFHSC standard case definitions: influenza-like illness. Falls Church, VA: Defense Health Agency, 2015.
2. Eick-Cost A, Hu Z. Relative effectiveness of cell-based influenza vaccines compared to egg-based influenza vaccines, active component US Service members, 2017-18 season. International Conference on Emerging Infectious Diseases. Atlanta, GA, 2018:54. Abstr. 129.

3. Eick-Cost AA, Hunt DJ. Assessment of ICD-9-based case definitions for influenza-like illness surveillance. MSMR 2015; 22(9): 2-7.

**Table S4. Subject demographics at baseline in the IIV3 cohort.**

| Characteristic | IIV3, 2017-2018 (n=174,191) | IIV3, 2018-2019 (n=106,670) |
|---|---|---|
| Mean age, years ± SD | 75.2 ± 6.9 | 75.4 ± 7 |
| Female sex, n (%) | 94,276 (54) | 57,358 (54) |
| Race and ethnicity, n (%) | | |
| White | 116,959 (67) | 72,536 (68) |
| Black or African American | 14,482 (8) | 8393 (8) |
| Other | 15,863 (9) | 9378 (9) |
| Race not reported | 26,887 (15) | 16,363 (15) |
| Hispanic ethnicity | 12,057 (7) | 6644 (6) |
| Geographic region, n (%) | | |
| Northeast | 33,021 (19) | 16,114 (15) |
| Midwest | 27,919 (16) | 19,843 (19) |
| South | 74,011 (42) | 48,159 (45) |
| West | 36,862 (21) | 21,038 (20) |
| Not Reported/Other | 2378 (1) | 1516 (1) |
| High-risk health condition | | |
| Chronic pulmonary disease | 49,947 (29) | 30,727 (29) |
| Myocardial infarction | 9317 (5) | 5994 (6) |
| Congestive heart failure | 17,275 (10) | 10,333 (10) |
| Cerebrovascular disease | 22,054 (13) | 14,290 (13) |
| Peripheral vascular disease | 30,126 (17) | 16,811 (16) |
| Renal disease | 28,051 (16) | 17,733 (17) |
| Diabetes not chronic | 49,946 (29) | 30,129 (28) |
| Diabetes chronic | 57,861 (33) | 34,804 (33) |
| Any malignancy | 26,346 (15) | 17,303 (16) |
| Metastatic tumor | 6937 (4) | 5248 (5) |
| AIDS/HIV | 468 (0) | 343 (0) |
| Rheumatic disease | 12,572 (7) | 7529 (7) |
| Mild liver disease | 10,508 (6) | 6268 (6) |
| Liver disease | 761 (0) | 405 (0) |
| Hemiplegia or paraplegia | 1487 (1) | 903 (1) |
| Dementia | 7607 (4) | 4860 (5) |
| Peptic ulcer disease | 4508 (3) | 2752 (3) |
| Charlson comorbidity index | 2.1 ± 1.5 | 2.2 ± 1.6 |

**Table S5. Adjusted rVE of aIIV3 versus comparators using AFHSC Code Set B to define influenza-related medical encounters in the 2017-2018 and 2018-2019 influenza seasons by age group.**

| | 2017-2018 Season rVE (95% CI) | | | 2018-2019 Season rVE (95% CI) | | |
|---|---|---|---|---|---|---|
| **Comorbidity** | **IIV4** | **HD-IIV3** | **IIV3** | **IIV4** | **HD-IIV3** | **IIV3** |
| Overall (≥1 health condition) | 7.1 (3.3 to 10.8) | -0.8 (-8.9 to 6.6) | -5.5 (-12.0 to 0.7) | 20.4 (16.2 to 24.4) | 2.7 (-2.7 to 7.8) | 8.0 (-29.0 to 34.4) |
| Chronic pulmonary disease | 3.7 (-3.3 to 10.2) | -2.8 (-17.4 to 10.1) | -3.6 (-14.3 to 6.1) | 15.5 (8.3 to 22.1) | -2.7 (-12.4 to 6.2) | 10.2 (-47.7 to 45.4) |
| Asthma | 4.9 (-5.4 to 14.2) | -1.3 (-23.0 to 16.5) | 0.7 (-16.1 to 15.1) | 17.4 (6.7 to 26.7) | -7.1 (-21.8 to 5.9) | 14.8 (-88.7 to 61.6) |
| Myocardial infarction or congestive heart failure | 5.7 (-5.8 to 16.0) | -7.5 (-35.5 to 14.6) | -9.5 (-25.3 to 4.1) | 16.8 (5.7 to 26.6) | 2.1 (-14.9 to 16.5) | 8.8 (-74.8 to 52.5) |
| Cerebrovascular disease or peripheral vascular disease | 2.1 (-6.6 to 10.0) | -8.0 (-27.4 to 8.4) | 1.1 (-9.5 to 10.7) | 18.9 (10.6 to 26.4) | 4.3 (-7.4 to 14.8) | 14.8 (-52.4 to 52.4) |
| Renal disease | 9.6 (-1.9 to 19.9) | -0.6 (-28.0 to 20.8) | -1.4 (-15.1 to 10.6) | 10.8 (-0.6 to 20.9) | -2.7 (-20.5 to 12.6) | -3.3 (-84.2 to 42.1) |
| Diabetes with or without complications | 6.9 (0.9 to 12.5) | -0.6 (-13.1 to 10.6) | -7.0 (-17.2 to 2.3) | 21.8 (16.0 to 27.3) | 4.0 (-4.4 to 11.7) | 12.0 (-49.0 to 48.0) |
| Any malignancy or metastatic tumor | 7.6 (-1.9 to 16.2) | -6.3 (-27.6 to 11.5) | -5.5 (-24.2 to 10.5) | 22.1 (9.2 to 33.3) | 3.8 (-9.3 to 15.2) | 5.7 (-119.3 to 59.4) |
| AIDS/HIV | 31.6 (-203.0 to 84.6) | 55.1 (-402.5 to 96.0) | -55.5 (-747.5 to 71.4) | 37.8 (-55.5 to 75.1) | 22.0 (-148.8 to 75.6) | -259.7 (-49,900.0 to 97.4) |
| Rheumatic disease | 4.1 (-9.9 to 16.5) | 1.8 (-26.9 to 24.0) | -8.6 (-34.4 to 12.4) | 14.3 (-3.8 to 29.3) | -2.6 (-21.8 to 13.6) | -4.2 (-240.1 to 68.1) |
| Liver disease | 1.6 (-19.0 to 18.6) | -10.5 (-60.0 to 23.7) | -3.6 (-27.6 to 15.8) | 5.3 (-16.1 to 22.8) | -10.7 (-40.8 to 12.9) | 9.9 (-156.4 to 68.4) |

Adjusted for age, sex, race, ethnicity, geographic region, week of influenza vaccination, and health status.
Abbreviations: aIIV3, adjuvanted trivalent inactivated influenza virus; CI, confidence interval; HD-IIV3, nonadjuvanted high-dose trivalent inactivated influenza virus; IIV4, nonadjuvanted quadrivalent inactivated influenza virus; IIV3, nonadjuvanted trivalent inactivated influenza virus; rVE, relative vaccine effectiveness.

**Table S6. Adjusted rVE of aIIV3 versus comparators using AFHSC Code Set A to define influenza in the 2017-2018 and 2018-2019 influenza seasons by age group.**

| | 2017-2018 Season rVE (95% CI) | | | 2018-2019 Season rVE (95% CI) | | |
|---|---|---|---|---|---|---|
| **Comorbidity** | **IIV4** | **HD-IIV3** | **IIV3** | **IIV4** | **HD-IIV3** | **IIV3** |
| Any comorbidity | 12.1 (11.0 to 13.3) | 2.3 (-0.2 to 4.9) | -2.6 (-4.6 to -0.6) | 16.6 (15.5 to 17.7) | 5.0 (3.8 to 6.4) | 4.8 (-3.8 to 12.7) |
| Chronic pulmonary disease | 8.0 (5.8 to 10.2) | -0.2 (-4.8 to 4.3) | -2.9 (-6.4 to 0.4) | 10.8 (8.8 to 12.7) | 2.5 (0.1 to 4.8) | 11.4 (-1.7 to 22.7) |
| Asthma | 7.8 (4.5 to 11.0) | -0.2 (-7.2 to 6.3) | -1.1 (-6.7 to 4.3) | 12.5 (9.5 to 15.5) | 3.3 (-0.1 to 6.5) | 13.2 (-9.1 to 30.9) |
| Myocardial infarction or congestive heart failure | 11.5 (7.9 to 15.0) | 2.8 (-5.4 to 10.3) | 1.4 (-3.2 to 5.8) | 14.6 (11.7 to 17.4) | 4.8 (0.8 to 8.6) | 4.1 (-13.9 to 19.3) |
| Cerebrovascular disease or peripheral vascular disease | 12.8 (10.3 to 15.3) | 2.3 (-3.5 to 7.6) | 2.3 (-1.2 to 5.7) | 13.3 (11.1 to 15.5) | 3.8 (1.0 to 6.5) | 5.8 (-10.0 to 19.2) |
| Renal disease | 12.4 (8.7 to 15.9) | 4.9 (-3.1 to 12.4) | -2.0 (-6.6 to 2.3) | 12.1 (9.3 to 14.9) | 3.9 (-0.3 to 7.8) | -0.8 (-17.0 to 13.0) |
| Diabetes with or without complications | 11.4 (9.5 to 13.3) | 2.5 (-1.5 to 6.3) | -0.3 (-3.3 to 2.7) | 16.4 (14.8 to 18.0) | 5.3 (3.3 to 7.3) | 5.8 (-8.3 to 18.2) |
| Any malignancy or metastatic tumor | 16.8 (14.1 to 19.4) | 2.5 (-3.5 to 8.2) | -4.7 (-10.4 to 0.7) | 21.5 (18.3 to 24.5) | 7.2 (4.4 to 10.1) | -0.6 (-24.7 to 18.7) |
| AIDS/HIV | -21.1 (-74.5 to 16.0) | -3.6 (-74.5 to 38.5) | 4.6 (-33.9 to 32.0) | 26.8 (6.9 to 42.5) | 16.9 (-11.1 to 37.9) | 12.2 (-83.2 to 57.9) |
| Rheumatic disease | 15.7 (11.5 to 19.6) | 6.2 (-2.7 to 14.3) | -0.7 (-8.3 to 6.4) | 19.8 (15.4 to 24.0) | 7.7 (3.2 to 11.9) | 3.3 (-33.3 to 29.9) |
| Liver disease | 7.4 (1.3 to 13.2) | 0.00 (-13.4 to 11.8) | -7.1 (-14.9 to 0.3) | 7.3 (2.3 to 12.2) | 3.6 (-2.7 to 9.3) | 4.1 (-27.6 to 28.0) |

Adjusted for age, sex, race, ethnicity, geographic region, week of influenza vaccination, and health status.
Abbreviations: aIIV3, adjuvanted trivalent inactivated influenza virus; CI, confidence interval; HD-IIV3, nonadjuvanted high-dose trivalent inactivated influenza virus; IIV4, nonadjuvanted quadrivalent inactivated influenza virus; IIV3, nonadjuvanted trivalent inactivated influenza virus; rVE, relative vaccine effectiveness.

**Figure S1. Standardized mean differences between aIIV3 and comparators.** (**a**) aIIV3 vs. IIV4, 2018. (**b**) aIIV3 vs. IIV4, 2019. (**c**) aIIV3 vs. HD-IIV3, 2018. (**d**) aIIV3 vs. HD-IIV3, 2019. (**e**) aIIV3 vs. IIV3, 2018. (**f**) aIIV3 vs. IIV3, 2019.

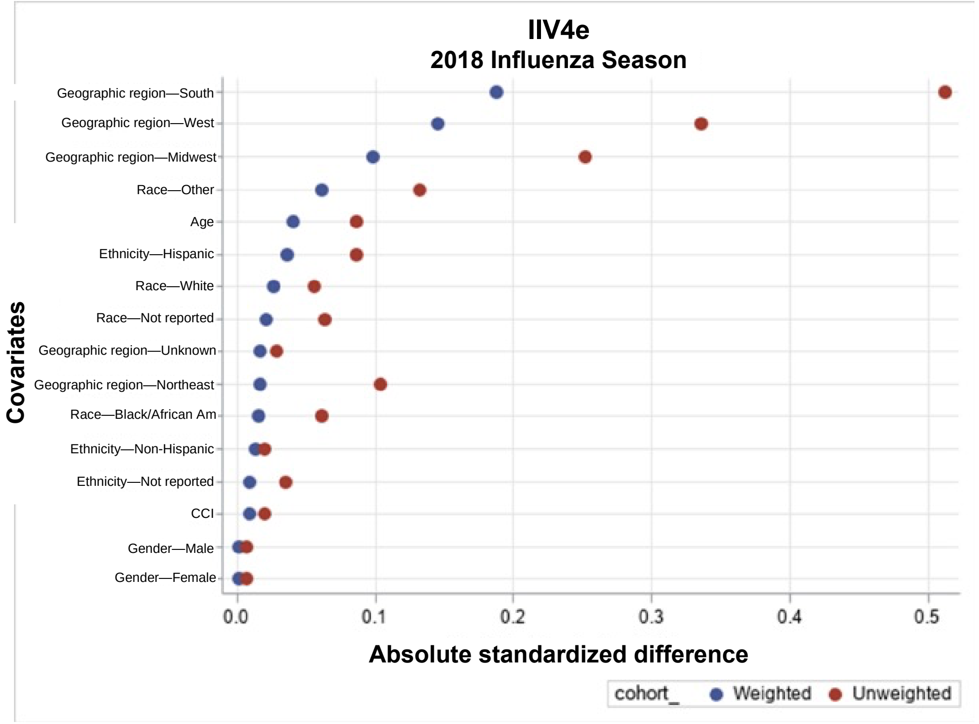


(**a**)

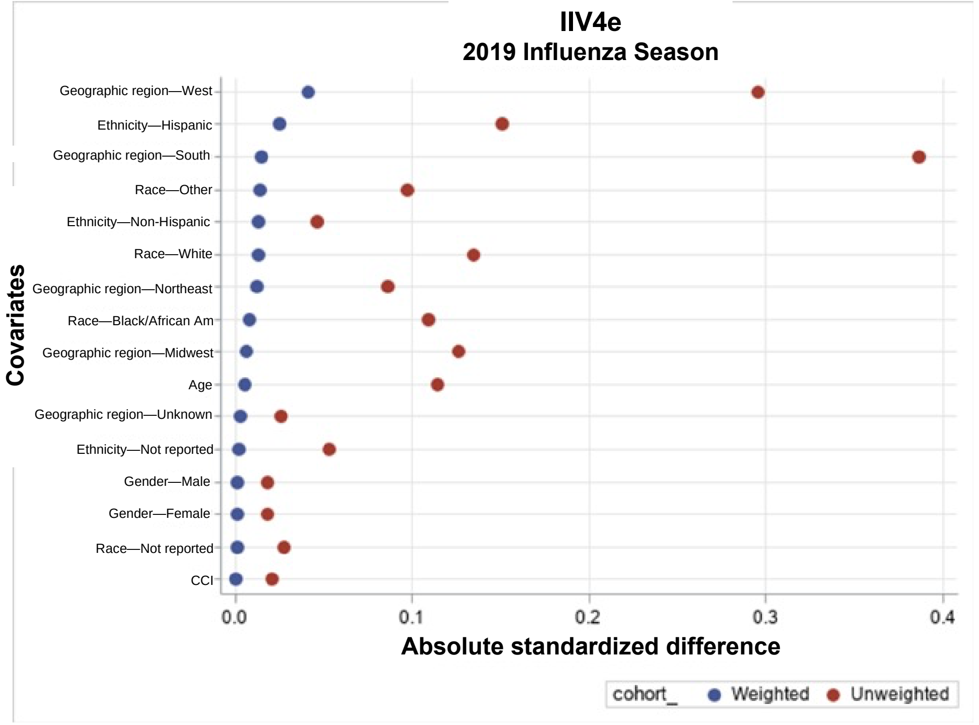


(**b**)

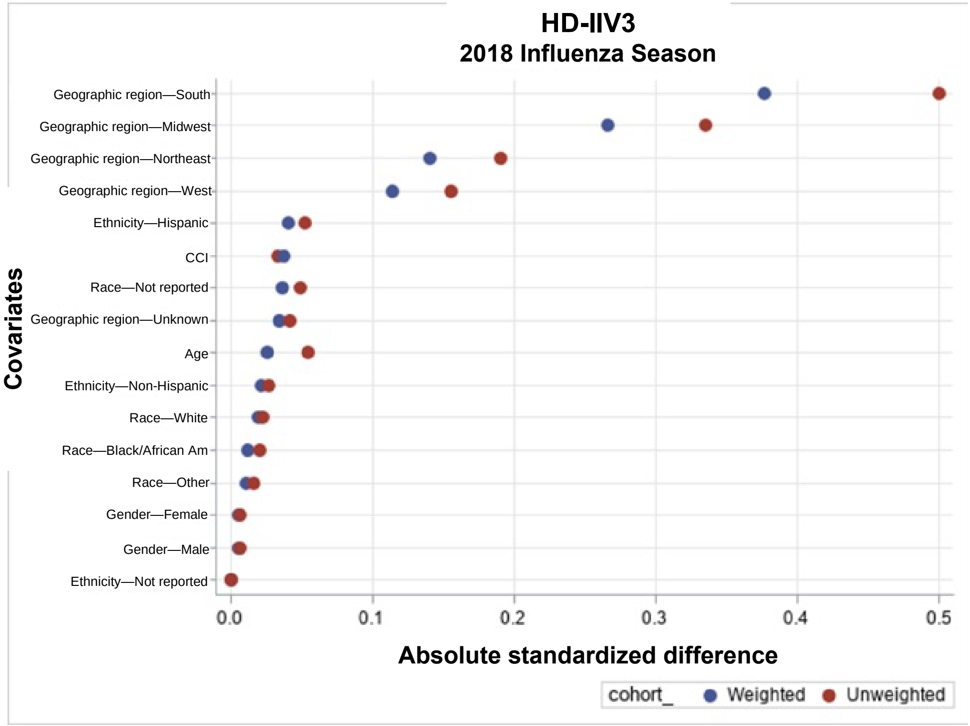
HD-IIV3
2018 Influenza Season
Geographic region—South
Geographic region—Midwest
Geographic region—Northeast
Geographic region—West
Ethnicity—Hispanic
CCI
Race—Not reported
Geographic region—Unknown
Age
Ethnicity—Non-Hispanic
Race—White
Race—Black/African Am
Race—Other
Gender—Female
Gender—Male
Ethnicity—Not reported
Covariates
0.0
0.1
0.2
0.3
0.4
0.5
Absolute standardized difference
cohort_
Weighted
Unweighted

(c)

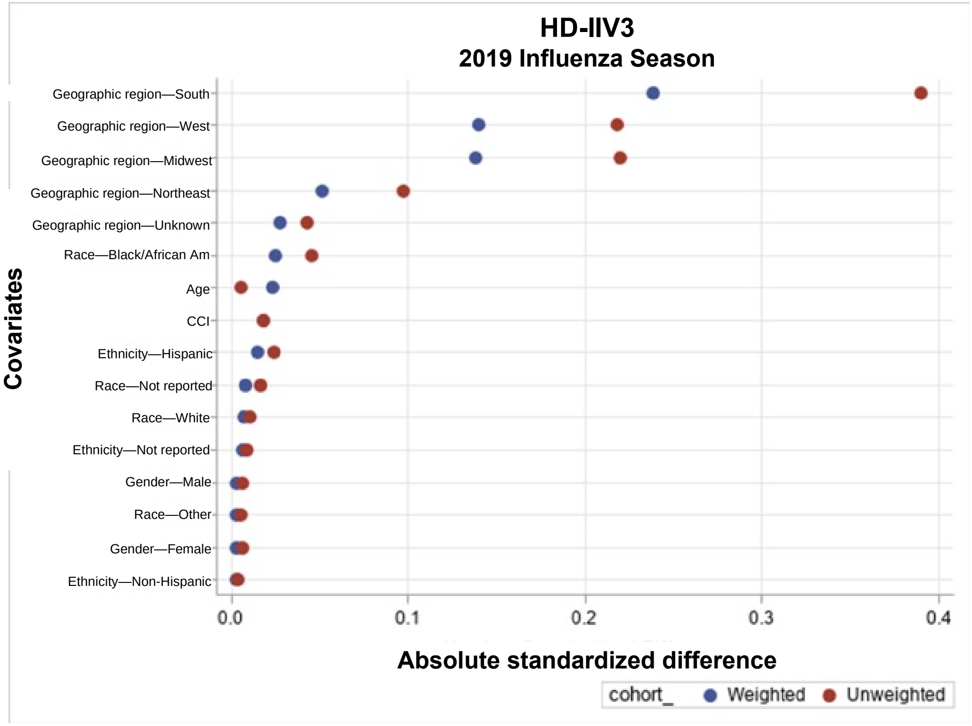
HD-IIV3
2019 Influenza Season
Geographic region—South
Geographic region—West
Geographic region—Midwest
Geographic region—Northeast
Geographic region—Unknown
Race—Black/African Am
Age
CCI
Ethnicity—Hispanic
Race—Not reported
Race—White
Ethnicity—Not reported
Gender—Male
Race—Other
Gender—Female
Ethnicity—Non-Hispanic
Covariates
0.0
0.1
0.2
0.3
0.4
Absolute standardized difference
cohort_
Weighted
Unweighted

(d)

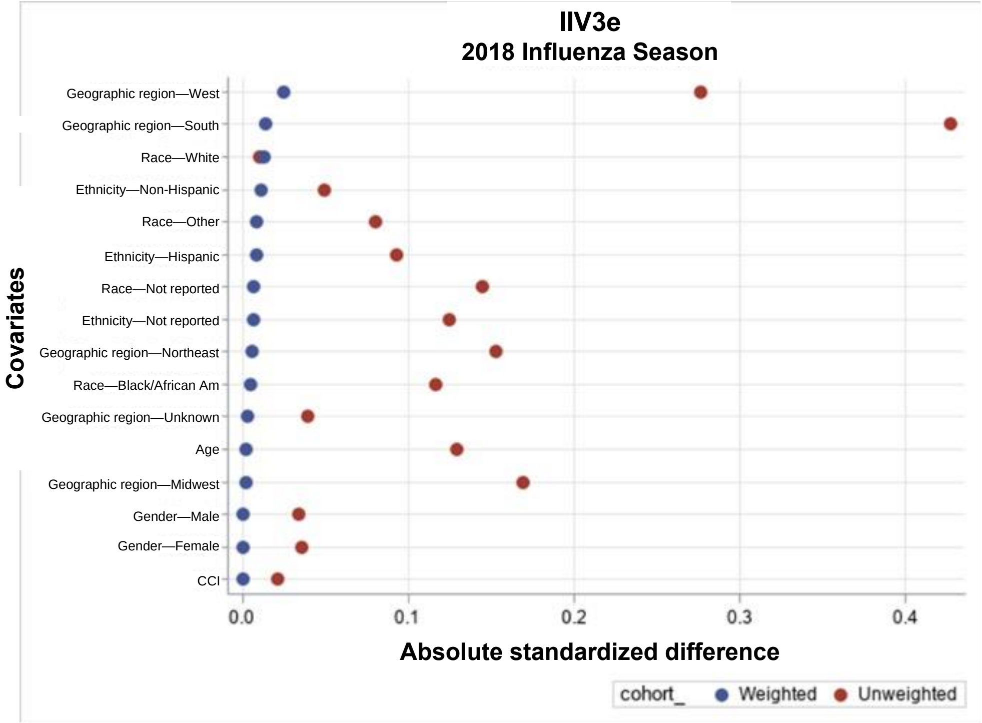
IIV3e
2018 Influenza Season
Covariates
Geographic region—West
Geographic region—South
Race—White
Ethnicity—Non-Hispanic
Race—Other
Ethnicity—Hispanic
Race—Not reported
Ethnicity—Not reported
Geographic region—Northeast
Race—Black/African Am
Geographic region—Unknown
Age
Geographic region—Midwest
Gender—Male
Gender—Female
CCI
0.0
0.1
0.2
0.3
0.4
Absolute standardized difference
cohort_
Weighted
Unweighted

(e)

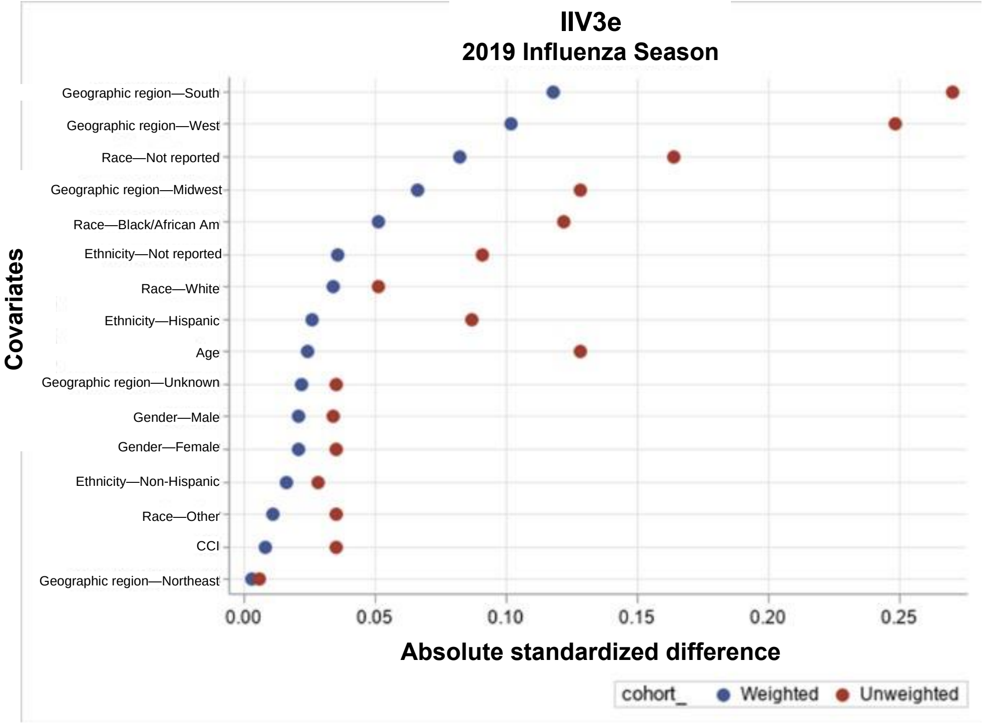
IIV3e
2019 Influenza Season
Covariates
Geographic region—South
Geographic region—West
Race—Not reported
Geographic region—Midwest
Race—Black/African Am
Ethnicity—Not reported
Race—White
Ethnicity—Hispanic
Age
Geographic region—Unknown
Gender—Male
Gender—Female
Ethnicity—Non-Hispanic
Race—Other
CCI
Geographic region—Northeast
0.00
0.05
0.10
0.15
0.20
0.25
Absolute standardized difference
cohort_
Weighted
Unweighted

(f)
